# Supplementary material for: A chemically inert bismuth interlayer enhances long-term stability of inverted perovskite solar cells
Source: Nat Commun. 2019 Mar 11;10:1161. doi: 10.1038/s41467-019-09167-0 (PMC6411982; doi:10.1038/s41467-019-09167-0)
Supplement: Supplementary file 1 — Supplementary Information [file 41467_2019_9167_MOESM1_ESM.pdf]

Supplementary Information

Wu et *al.* A chemically inert bismuth interlayer enhances long-term stability of inverted perovskite solar cells

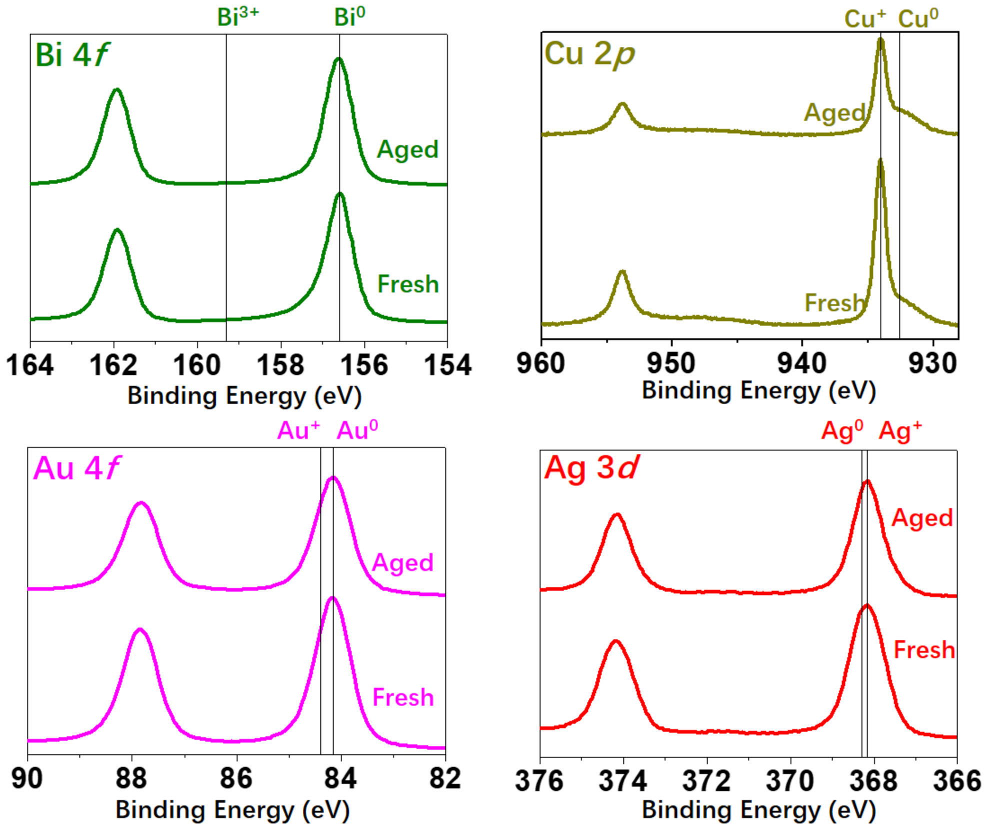

Supplementary Figure 1. XPS spectra of fresh and aged $MAPbI_3$/metal samples, which were prepared by evaporating 5-nm-thick Bi, Cu, Au or Ag films (which should have island-like morphology) directly onto $MAPbI_3$ films. Aging condition: dark, N_2 atmosphere, 85 ℃, 100 hours.

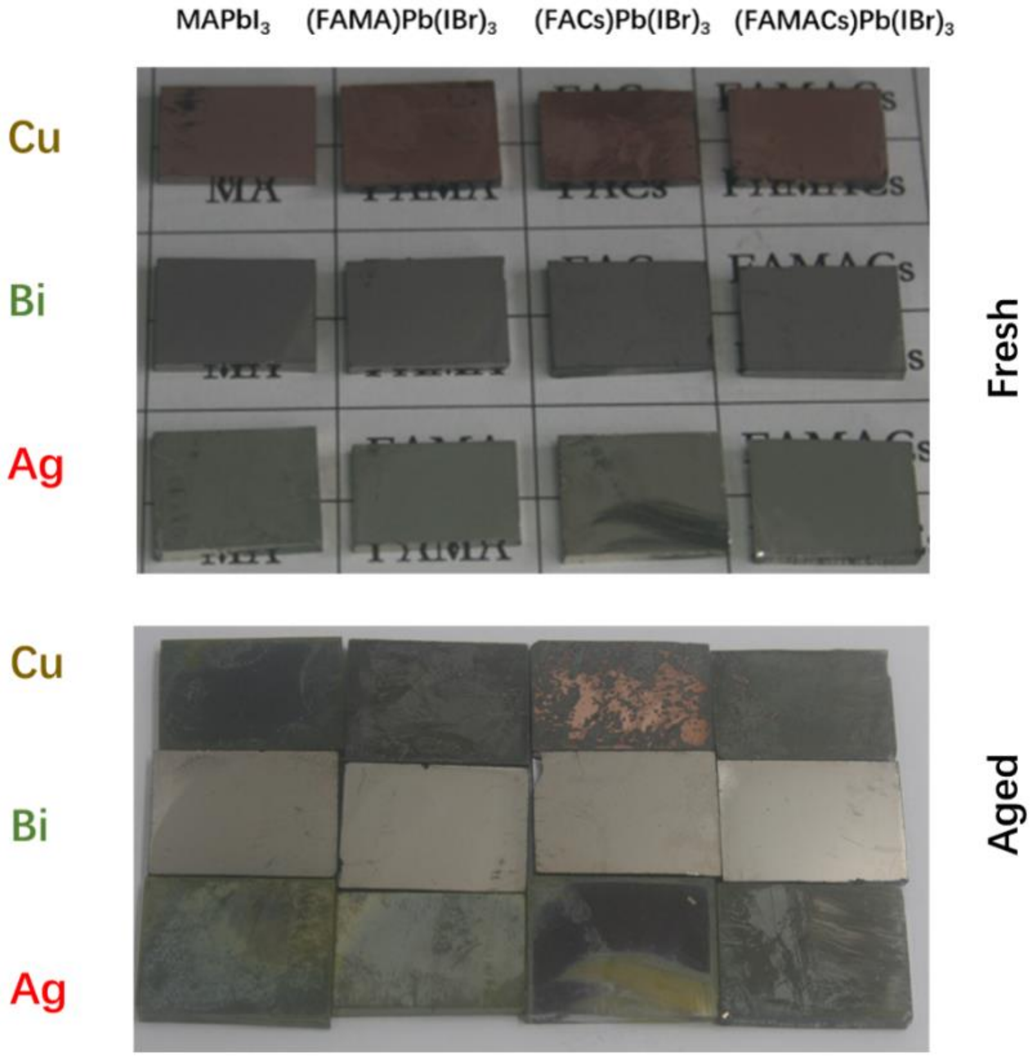

Supplementary Figure 2. Photos of samples prepared by evaporating 40 nm-thick Cu, Ag, and Bi films onto $MAPbI_3$, $(FAMA)Pb(IBr)_3$, $(FACs)Pb(IBr)_3$, and $(FAMACs)Pb(IBr)_3$ films before and after thermal aging. Aging condition: dark, ambient air, 85 °C, 48 hours.

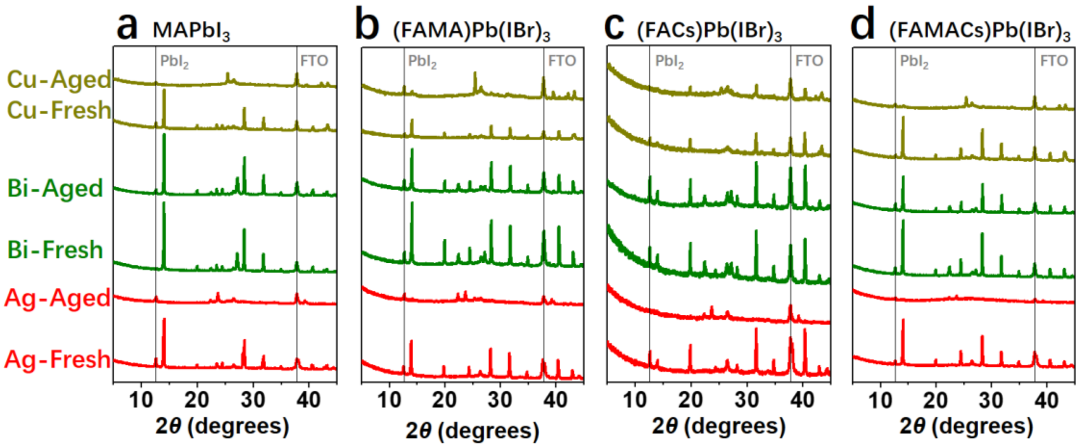

Supplementary Figure 3. XRD spectra of samples prepared by evaporating 40 nm-thick Cu/Ag/Bi films onto $MAPbI_3$, $(FAMA)Pb(IBr)_3$, $(FACs)Pb(IBr)_3$, and $(FAMACs)Pb(IBr)_3$ films before and after thermal aging. Aging condition: dark, ambient air, 85 °C, 48 hours.

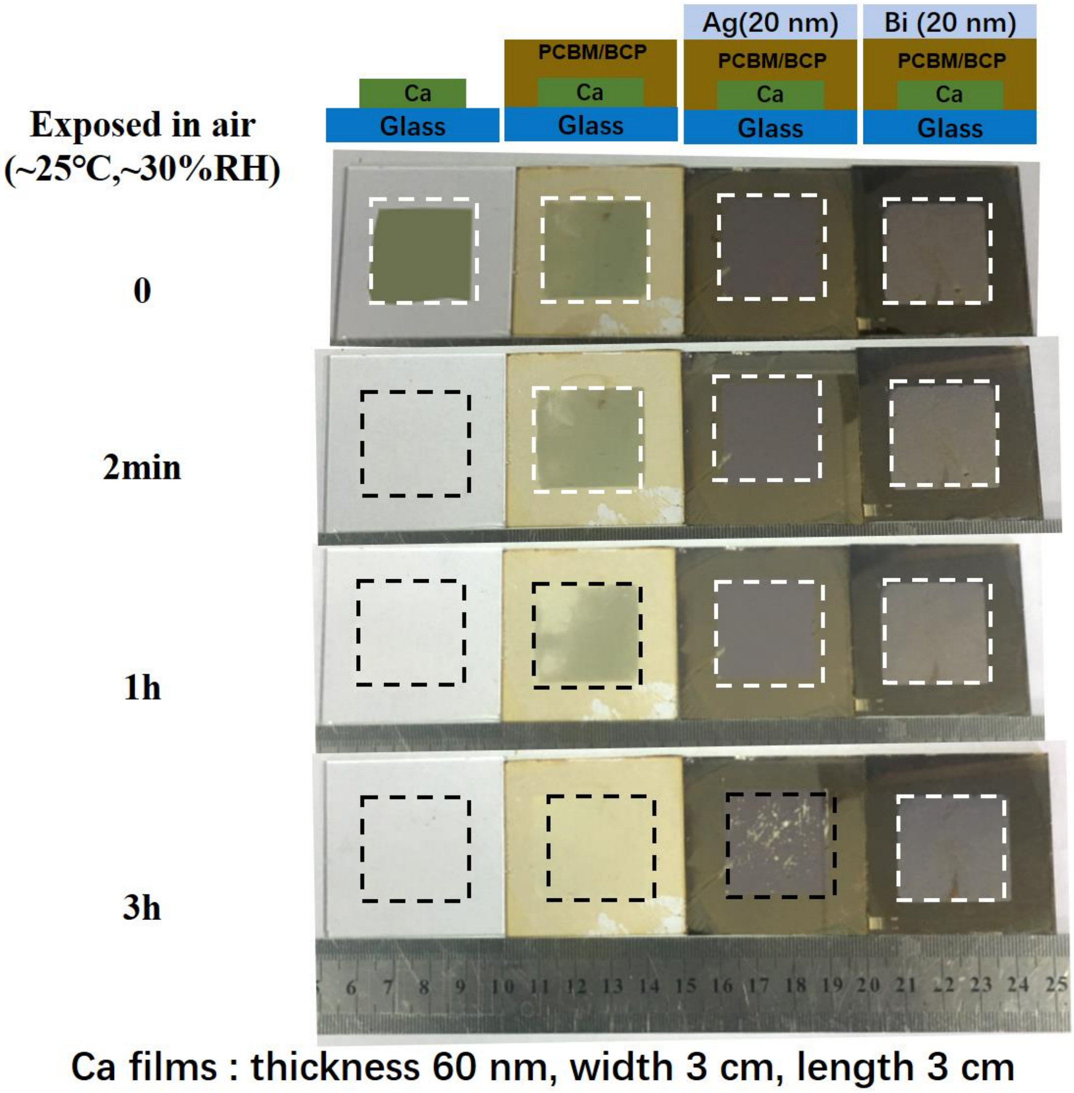

Supplementary Figure 4. Water/oxygen vapor transmission rate test. Samples include the thermal-evaporated Ca films (60 nm thick, 3×3 cm^2 area) on glass substrates, as well as that covered by different interfacial layers, including PCBM (60 nm)/BCP (5 nm), PCBM (60 nm)/BCP (5 nm)/Ag (20 nm) and PCBM (60 nm)/BCP (5 nm)/Bi (20 nm). The test was conducted in controlled air (25 ℃ and 30% RH) and the pictures were taken from glass side.

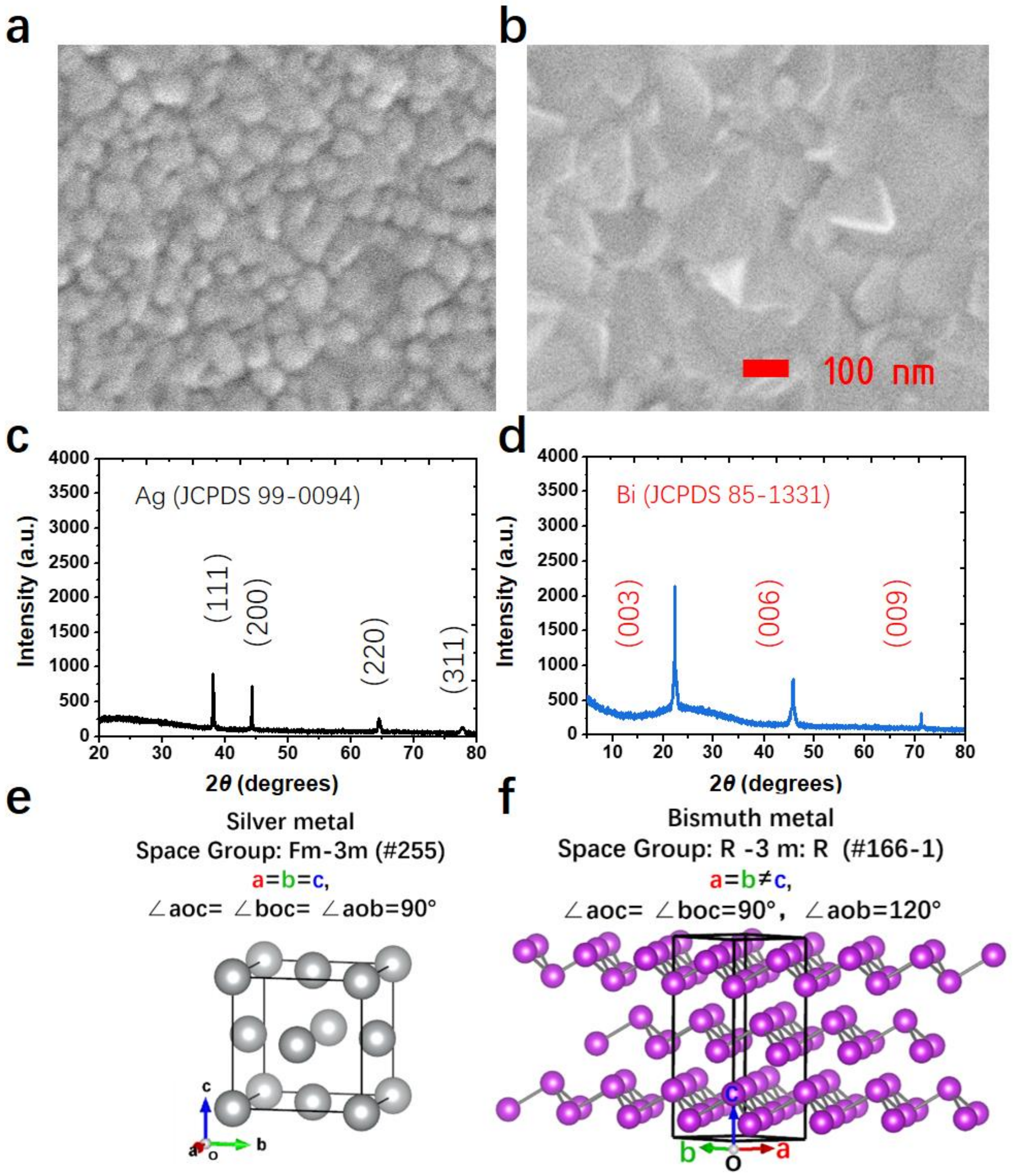

Supplementary Figure 5. Comparison of the as-deposited Ag film (20 nm) and Bi film (20 nm): (**a**-**b**) SEM images of Ag and Bi films on glass/PCBM/BCP substrates (scale bar: 100 nm), (**c**-**d**) XRD patterns of Ag and Bi films on Glass/PCBM/BCP substrates, and (**e**-**f**) crystal structure diagram of Ag and Bi.

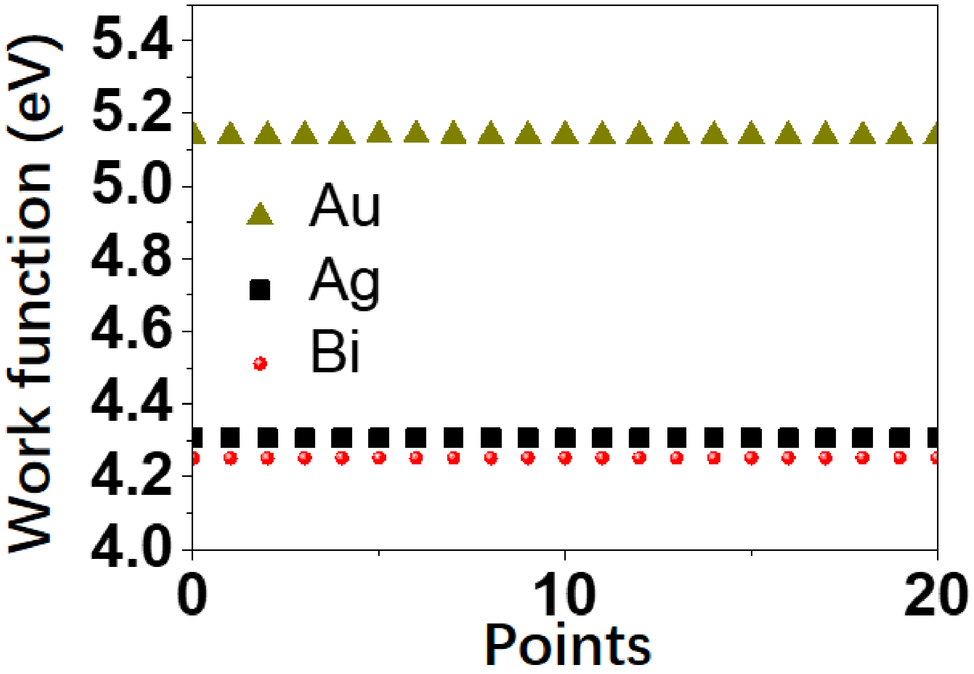

Supplementary Figure 6. The work functions (WF) of Au, Ag and Bi. Before measuring, the WF of the tip was calibrated by a standard golden specimen with the WF of 5.15 eV.

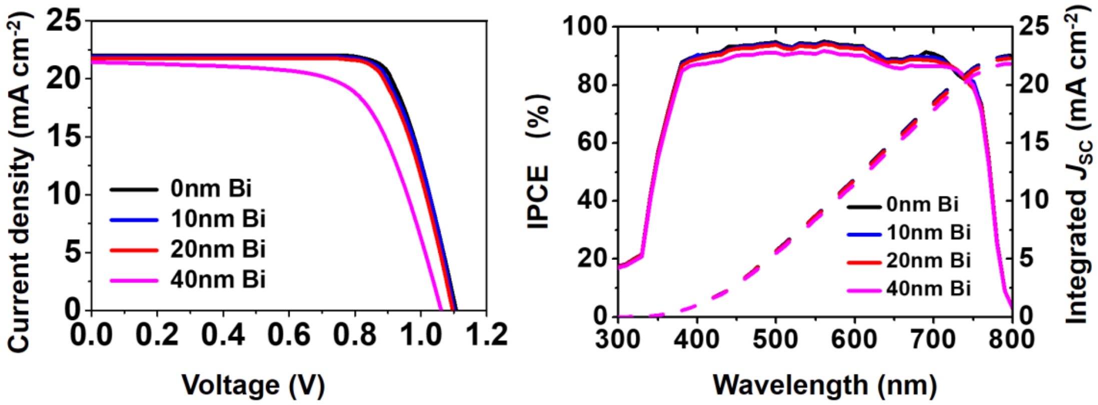

Supplementary Figure 7. *J*-*V* curves and IPCEs of large-area (1 cm^2) MA-HPVKSCs with different thicknesses of Bi interlayers. Scanning mode: forward scan (from -0.1 V to 1.2 V).

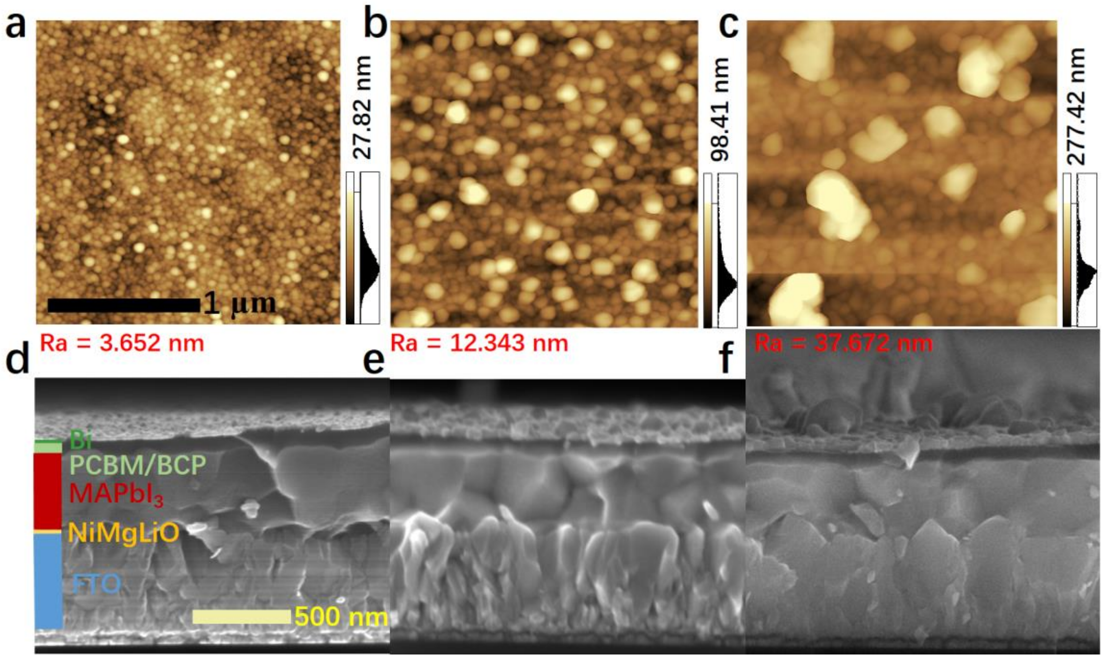

Supplementary Figure 8. AFM (scale bar: 1 μm) and SEM (scale bar: 500 nm) cross-sectional images of the Bi layers (on the $MAPbI_3$/PCBM/BCP substrates) with the thicknesses of 10 nm (**a**, **d**), 40 nm (**b**, **e**), and 80 nm (**c**, **f**).

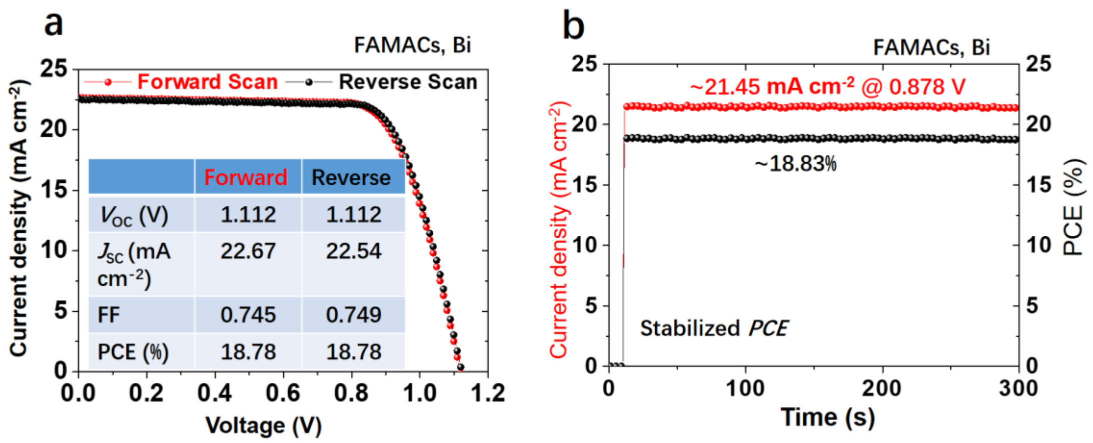

Supplementary Figure 9. *J*-*V* curves and the corresponding Stabilized PCE of a typical FAMACs-HPVKSC (1 cm^2) with optimized Bi interlayer under AM 1.5 G simulated sunlight (100 mW cm^{-2}).

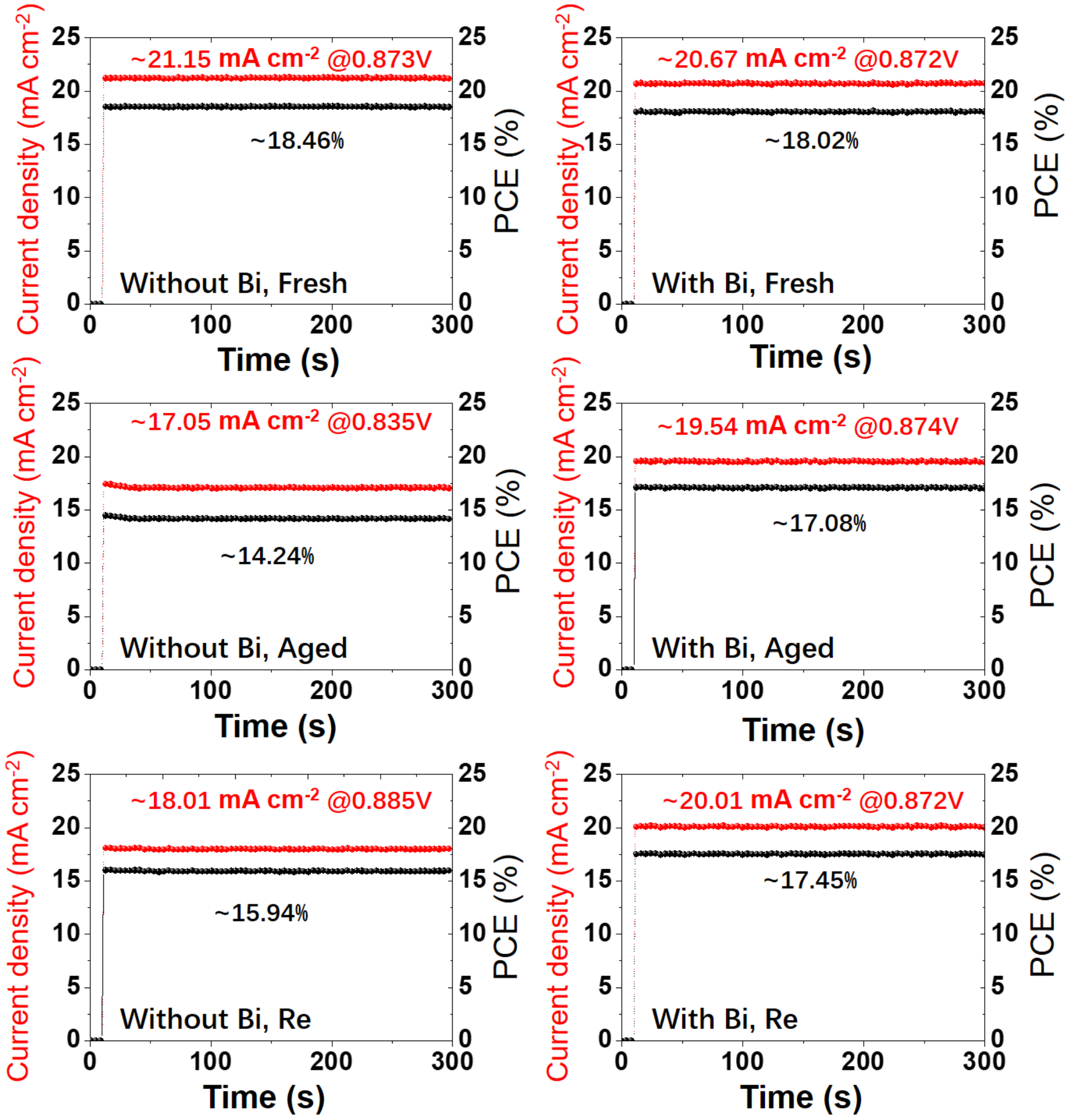

Supplementary Figure 10. Changes of stabilized PCEs for the MA-HPVKSCs without and with Bi interlayers, including the fresh devices, aged devices and devices constituted of the aged $MAPbI_3$ films with re-prepared top layers. Aging conditions: 85 ℃, dark and N_2 atmosphere for 100 hours.

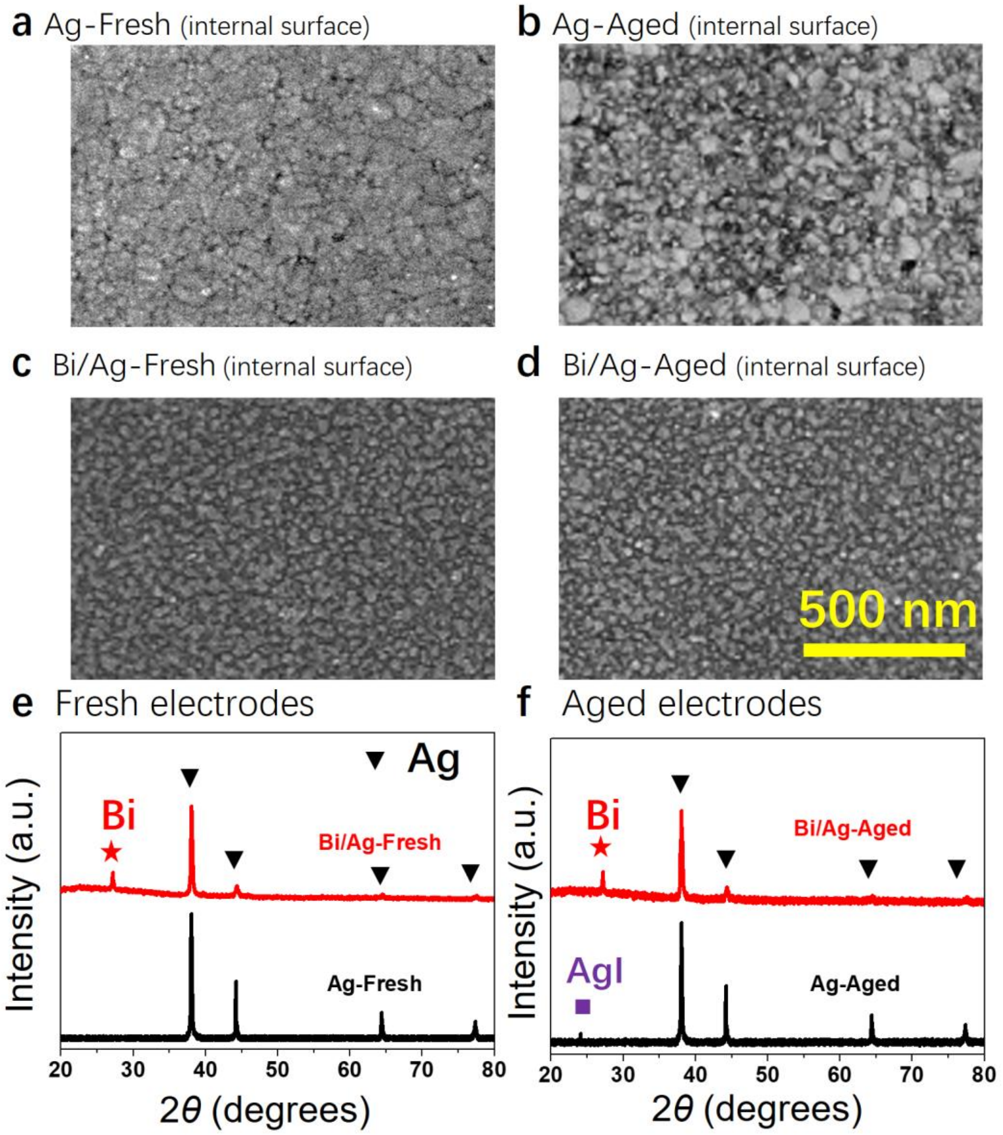

Supplementary Figure 11. Changes of the electrode layers during thermal aging. (**a-d**) SEM images of the metal electrodes' inner surfaces before and after aging at 85 ℃ for 100 h in a N_2 filled glovebox in the dark. (scale bar: 500 nm) (**a**) and (**b**) are the sole Ag electrodes, (**c**) and (**d**) are the Bi/Ag electrodes. The metal electrode samples were obtained by dissolving PCBM/BCP in MA-HPVKSCs using chlorobenzene. (**e**) and (**f**) are XRD patterns of the inner surfaces of Ag and Bi/Ag electrodes peeled from the fresh and aged devices.

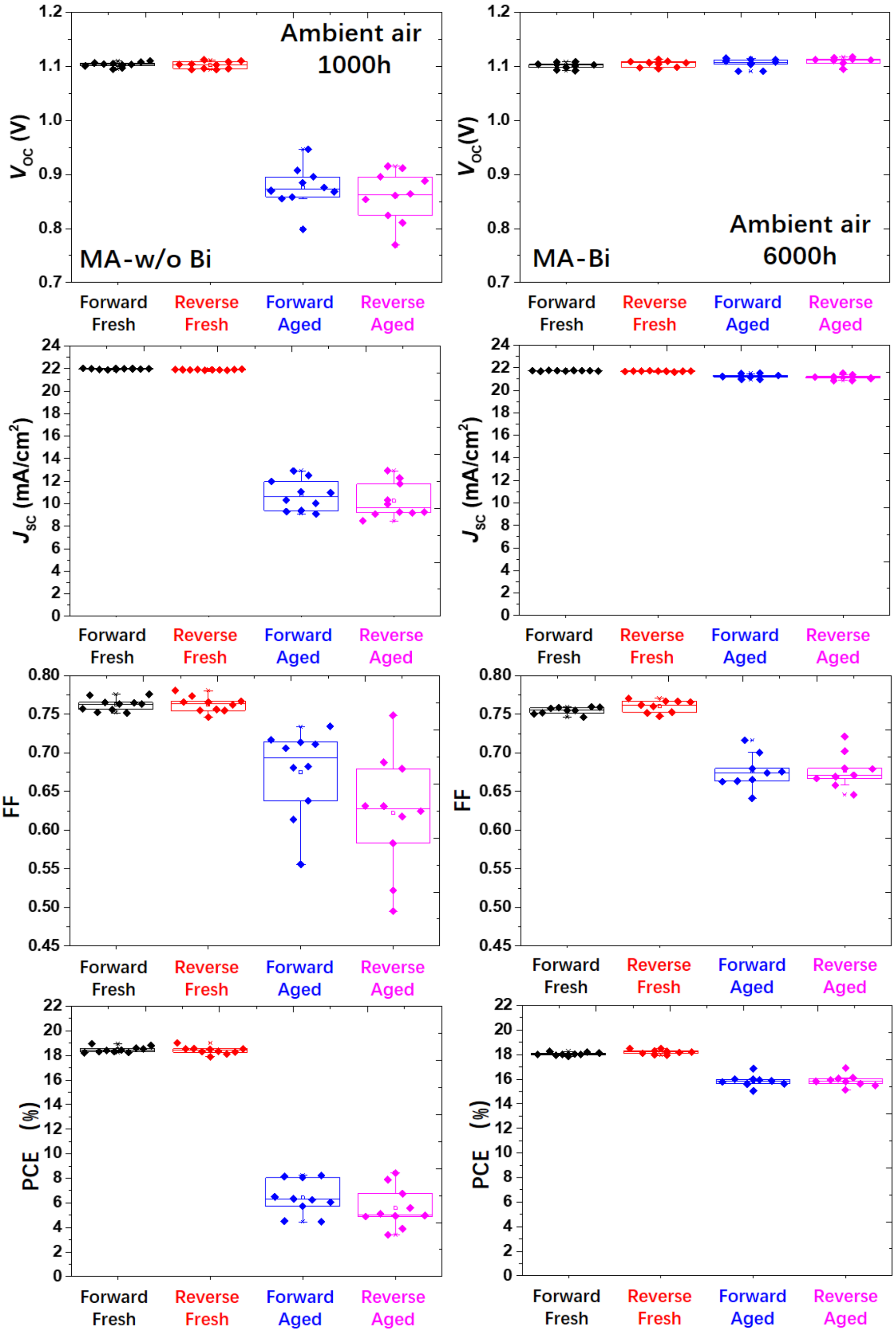

Supplementary Figure 12. Performance statistics of MA-HPVKSCs with (9 cells) and without (10 cells) Bi before and after long-term storage aging. Devices were stored in the dark under ambient air at RT without humidity control for 6000 hours, and their *J-V* curves were tested in ambient air periodically. Forward Scan: from -0.1 V to 1.2 V. Reverse Scan: from 1.2 V to -0.1 V. Light source: standard AM1.5G simulated sunlight.

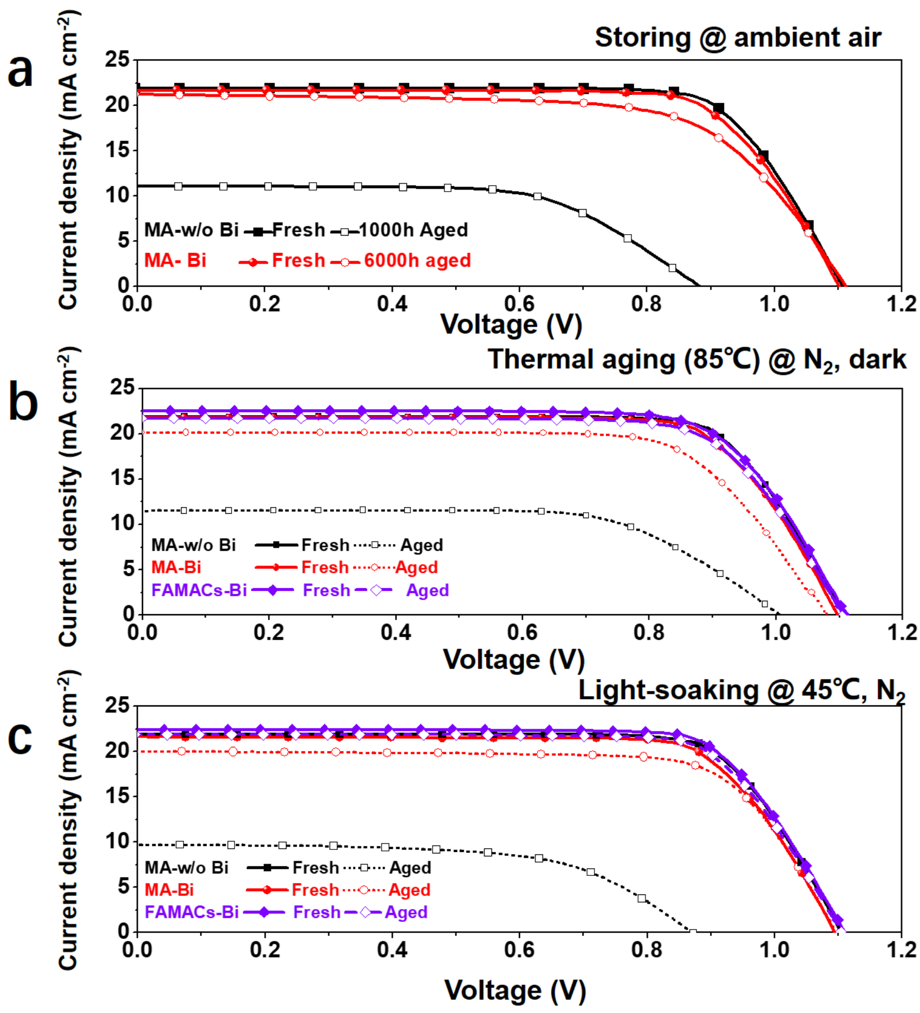

Supplementary Figure 13. *J-V* curves of the un-encapsulated large-area (1 cm^2) HPVKSCs with and without Bi interlayer under different aging conditions, including (**a**) ambient air storage in the dark without humidity control, (**b**) 85 ℃ thermal aging in N_2 atmosphere, and (**c**) light-soaking under continuous illumination in N_2 atmosphere, near maximum power electrical biases and with the cell temperature of 45 ℃ after equilibrium. Scanning mode: forward scan (from -0.1 V to 1.2 V). Light source in (**a-b**): standard AM1.5G simulated sunlight. Light source in (**c**): a white light LED array with light intensity calibrated to achieve the same J_{SC} as for 1 sun AM1.5G solar irradiation.

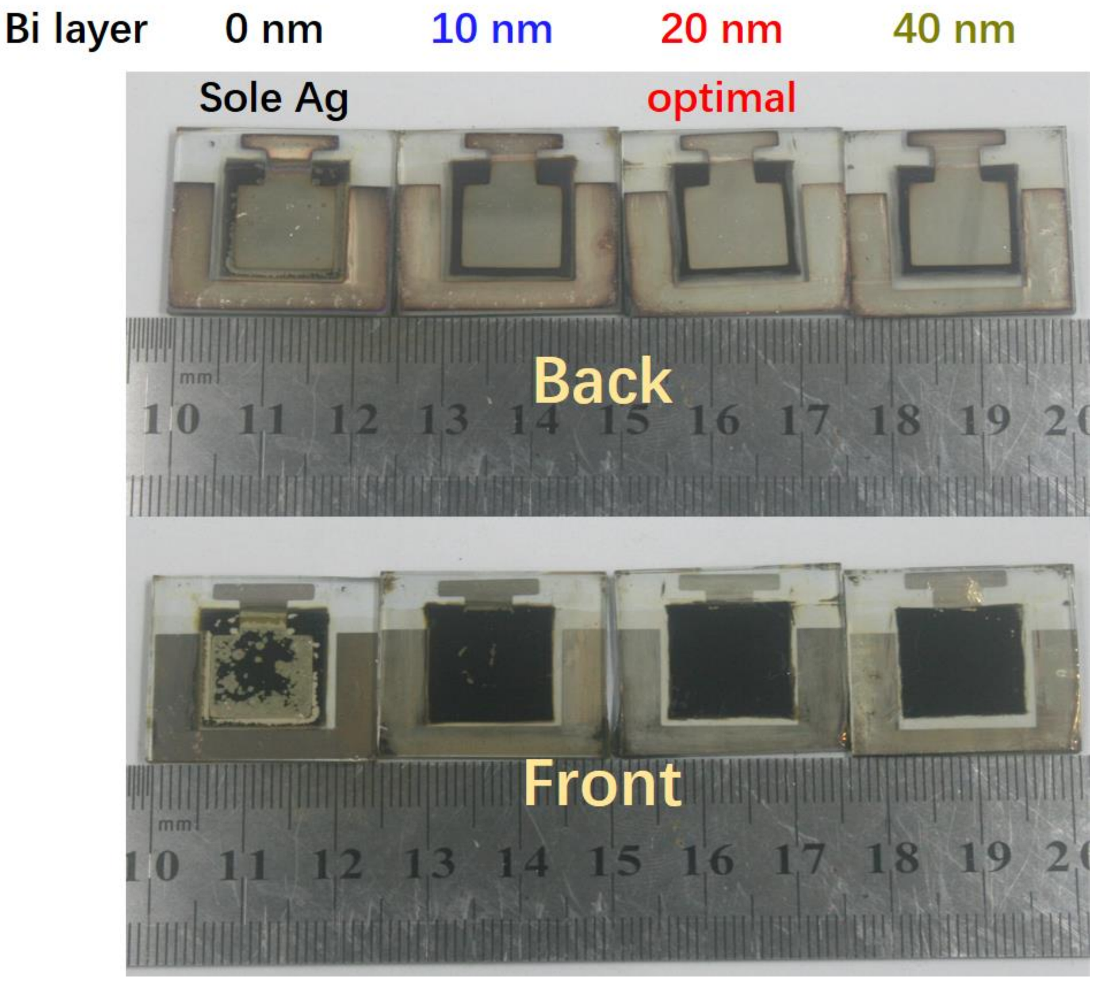

Supplementary Figure 14. Optical photos of aged devices with different thicknesses of Bi interlayers after exposure to ambient air (relative humidity of the local climate is normally 40 to 90 %RH) for 6000 hours without encapsulation.

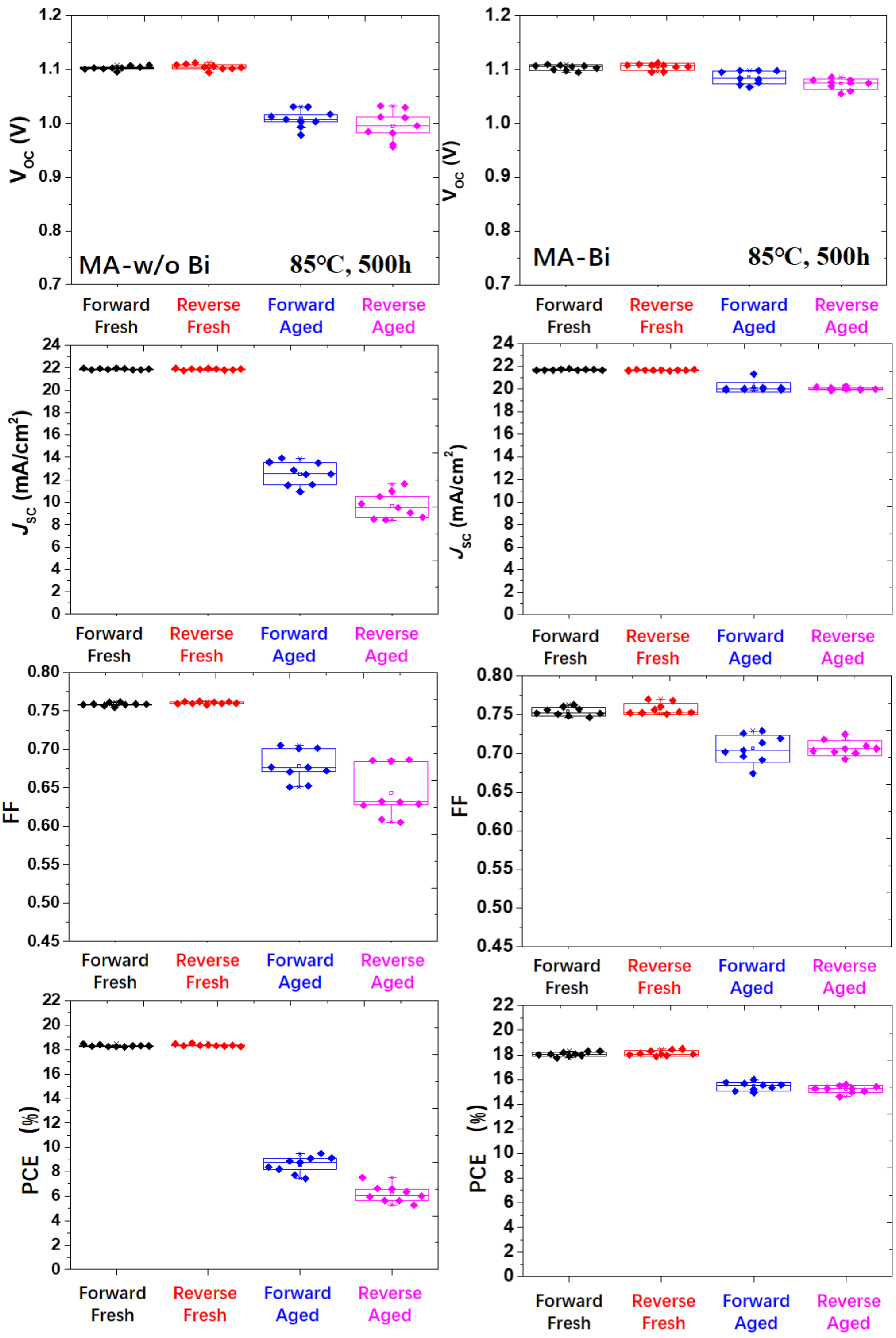

Supplementary Figure 15. Performance statistics of MA-HPVKSCs with (9 cells) and without (9 cells) Bi before and after thermal aging (85 ℃) in the dark under N_2 atmosphere for 500 h. Forward Scan: from -0.1 V to 1.2 V. Reverse Scan: from 1.2 V to -0.1 V. Light source: standard AM1.5G simulated sunlight.

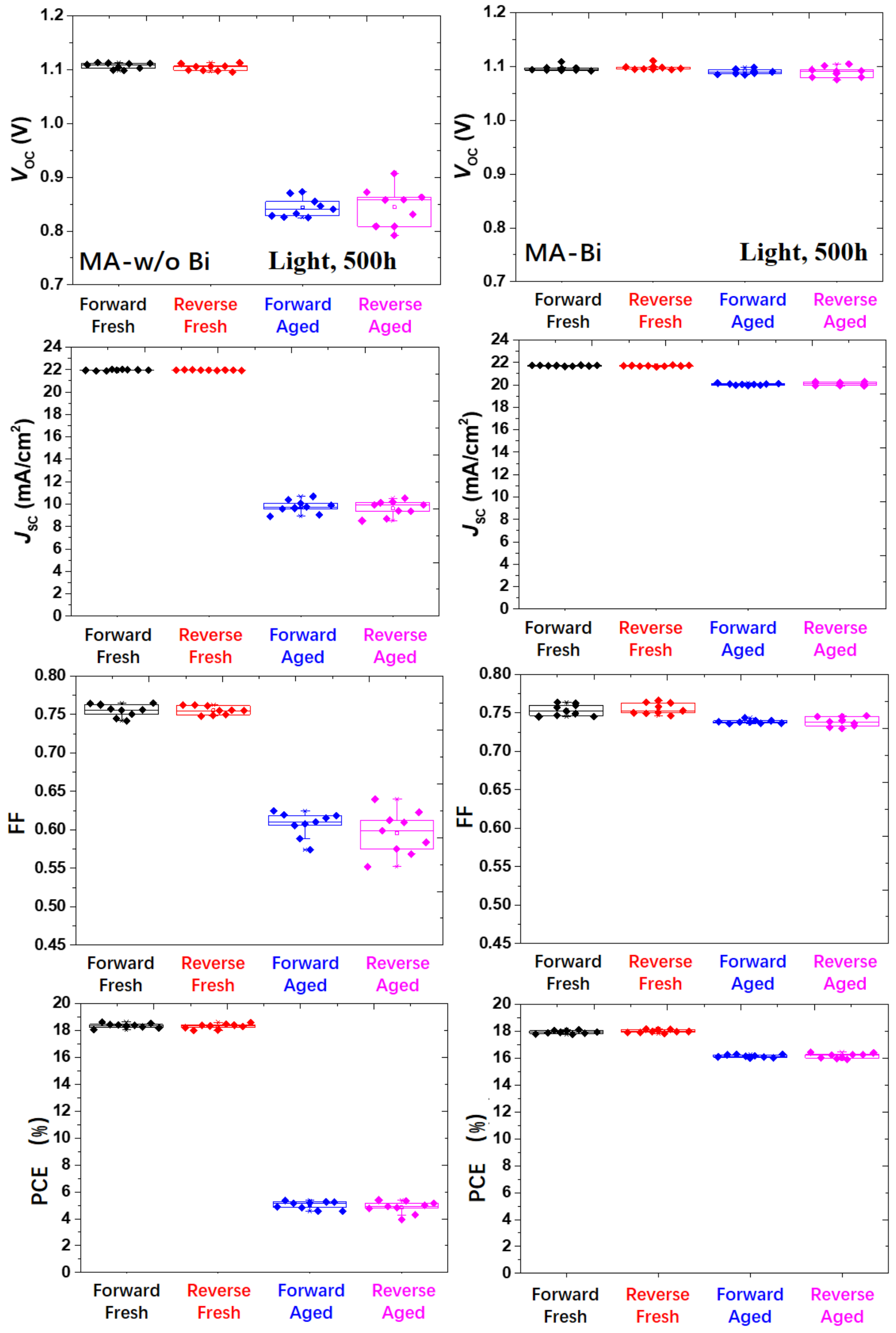

Supplementary Figure 16. Performance statistics of MA-HPVKSCs with (9 cells) and without (9 cells) Bi before and after light-soaking for 500 hours in N_2 atmosphere, near maximum power electrical biases and with the cell temperature of 45 ℃ after equilibrium. The performance of devices was determined under white LED light, of which the light intensity was calibrated to achieve the same J_{SC} as for 1 sun AM1.5G solar irradiation. Forward Scan: from -0.1 V to 1.2 V. Reverse Scan: from 1.2 V to -0.1 V.

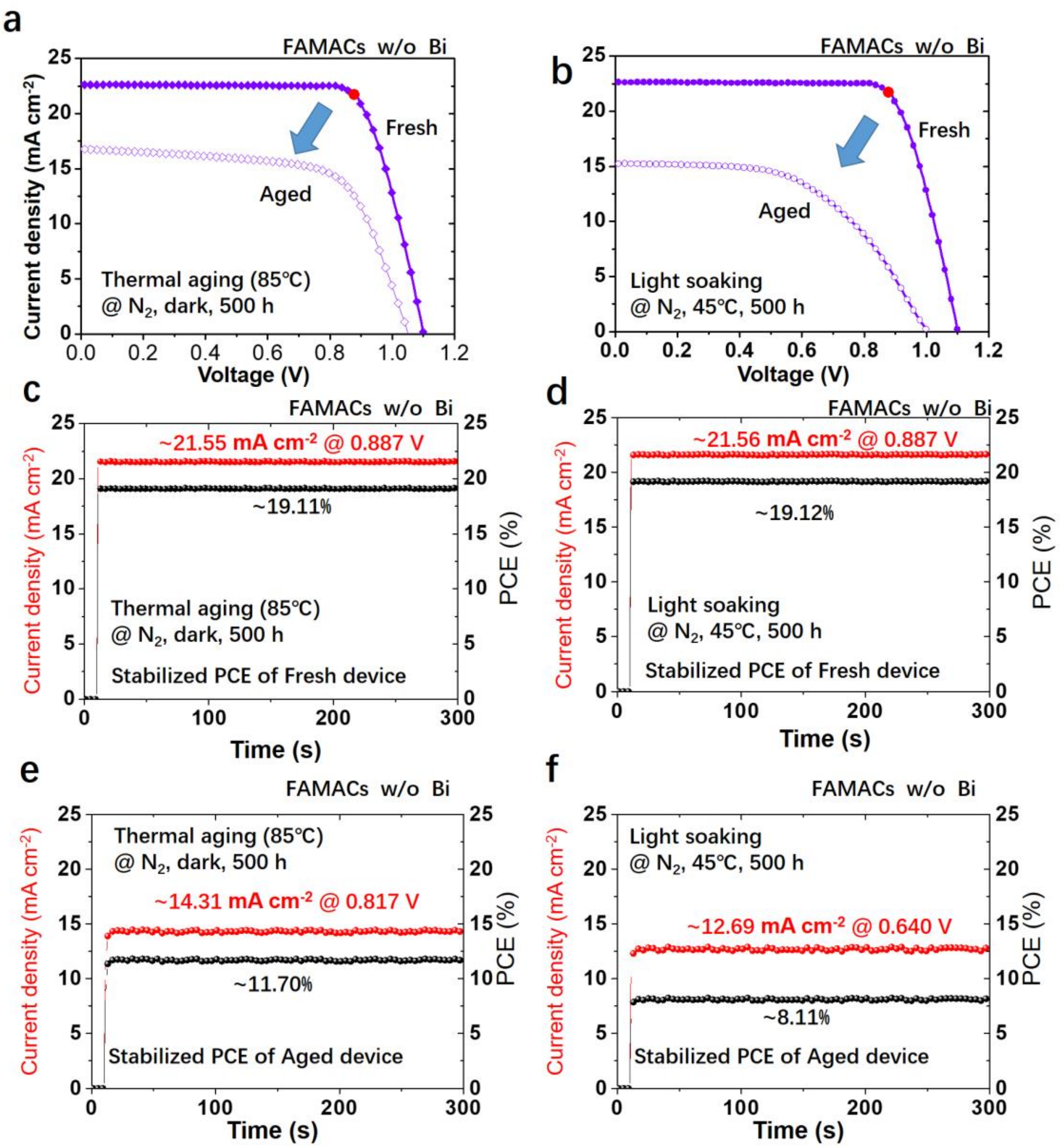

Supplementary Figure 17. Performance of the un-encapsulated large-area (1 cm^2) FAMACs-HPVKSCs without Bi interlayer before and after aging under different aging conditions. (**a**) *J-V* curves of a typical FAMACs-HPVKSC without Bi before and after thermal aging (85 ℃) in N_2 atmosphere for 500 h. (**b**) *J-V* curves of a typical FAMACs-HPVKSC without Bi before and after aging under continuous illumination in N_2 atmosphere, near maximum power electrical biases and with the cell temperature of 45 ℃ after equilibrium. The light intensity for aging was generated by a white light LED array and calibrated to achieve the same J_{SC} of HPVKSCs as upon 1 sun AM1.5G solar irradiation. (**c-f**) The corresponding fresh FAMACs-HPVKSCs without Bi showed stabilized PCEs of over 19.1% under standard AM1.5G simulated sunlight and white LED light with calibrated intensity as the same as 1 sun AM1.5G solar irradiation.

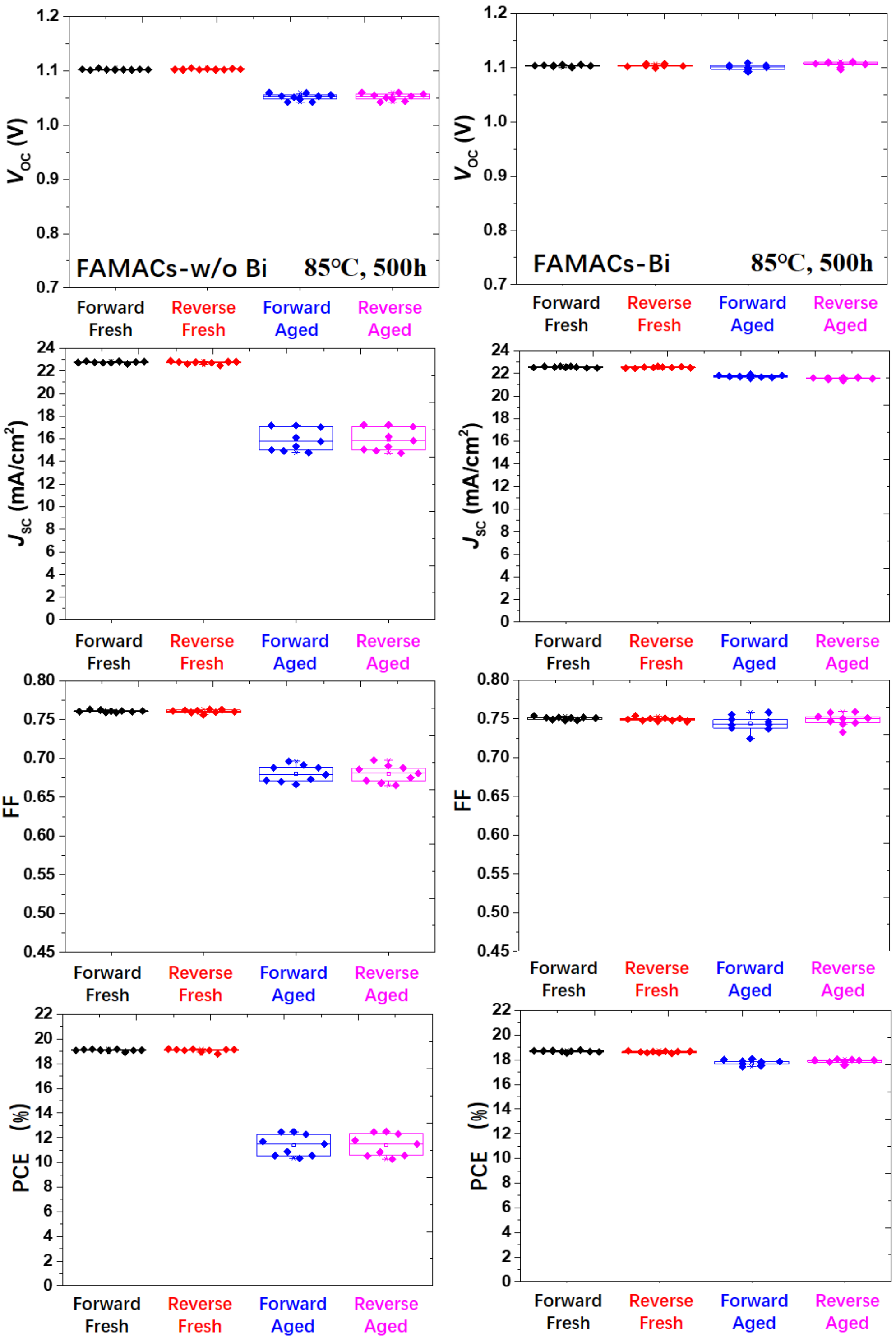

Supplementary Figure 18. Performance statistics of FAMACs-HPVKSCs with (9 cells) and without (9 cells) Bi before and after thermal aging (85 ℃) in the dark under N_2 atmosphere for 500 h. Forward Scan: from -0.1 V to 1.2 V. Reverse Scan: from 1.2 V to -0.1 V. Light source: standard AM1.5G simulated sunlight.

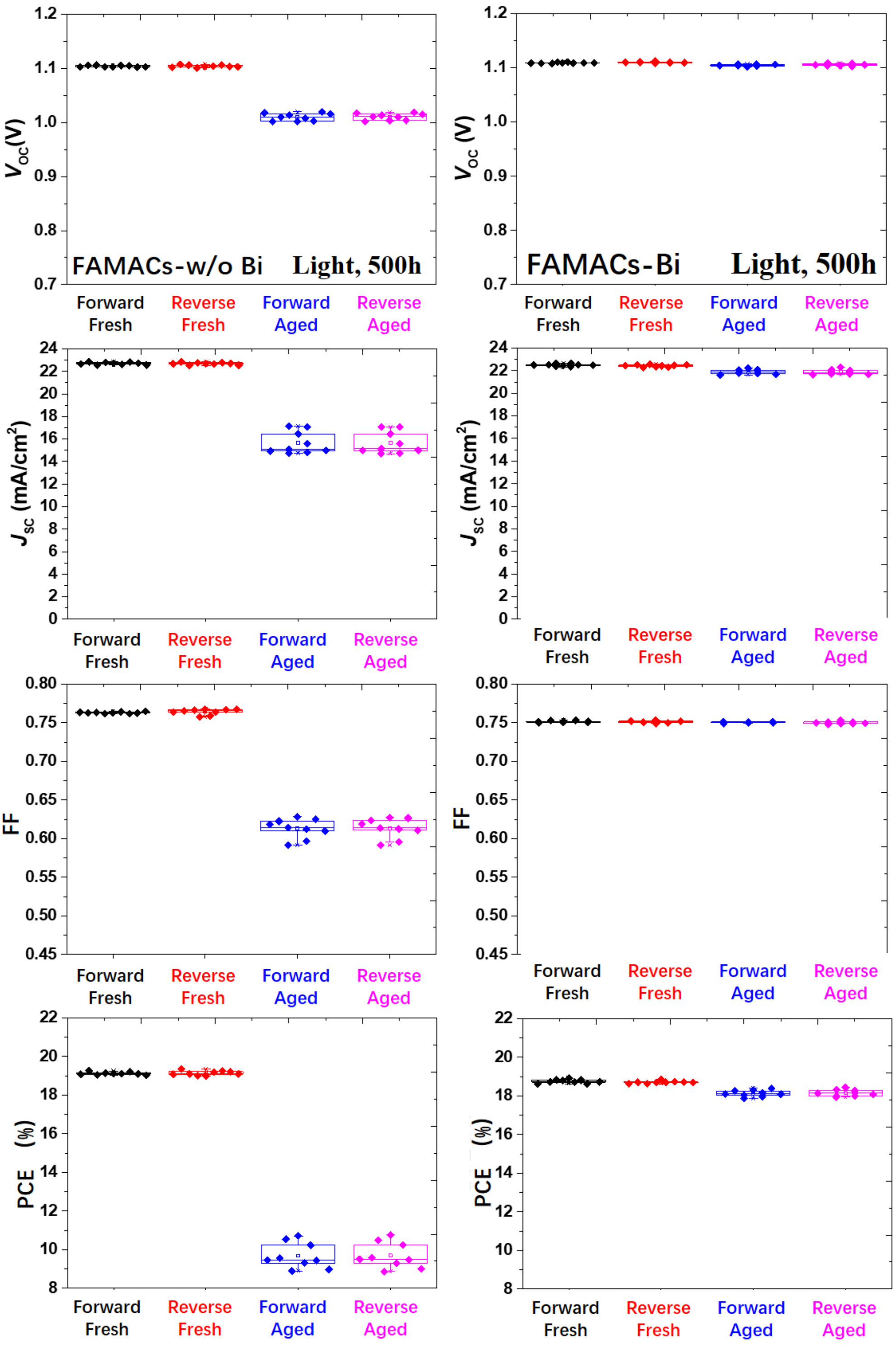

Supplementary Figure 19. Performance statistics of FAMACs-HPVKSCs with (9 cells) and without (9 cells) Bi before and after light-soaking for 500 hours in N_2 atmosphere, near maximum power electrical biases and with the cell temperature of 45 ℃ after equilibrium. The performance of devices was determined under white LED light, of which the light intensity was calibrated to achieve the same J_{SC} as upon 1 sun AM1.5G solar irradiation. Forward Scan: from -0.1 V to 1.2 V. Reverse Scan: from 1.2 V to -0.1 V.

Supplementary Table 1. Summary of the bond energies and standard enthalpies for common elements. [1-3]

Material	Metal - Metal ($E_{M\text{-}M}$) (kJ/mol)	Metal -Iodine ($E_{M\text{-}I}$) (kJ/mol)	Energy barrier ($\Delta E = E_{M\text{-}M} - E_{M\text{-}I}$) (kJ/mol)	Standard enthalpy of formation ($\Delta_f H$, 298.15K) (kJ/mol)
**Ag**	162.9	234	-71.1	-61.8 (AgI)
**Al**	264.3	369.9	-105.6	-302.9 (AlI_3)
**Au**	226.2	276	-49.8	0 (AuI)
**Bi**	204.4	186.1	18.3	-150.69 (BiI_3)
**C**	618.3	253.1	365.2	-392.9 (CI_4)
**Mo**	435.5	266.9	168.6	-103.9 (MoI_2)
**Cd**	7.36	97.2	-89.84	-203.3 (CdI_2)
**Co**	127	280	-153	-88.7 (CoI_2)
**Cr**	152	287	-135	-205.0 (CrI_3)
**Cu**	201	289	-88	-68.7 (CuI)
**Fe**	118	123	-5	-113 (FeI_2)
**Sn**	187	235	-48	-143.5 (SnI_2)
**In**	82.0	306.9	-224.9	-238.0 (InI_3)
**Ni**	204	293	-89	-78.2 (NiI_2)
**Ti**	117.6	306	-188.4	-375.7 (TiI_4)
**Mg**	11.3	229	-217.7	-364.0 (MgI_2)
**Zn**	22.2	153.1	-130.9	-208.0 (ZnI_2)
**Pb**	86.6	194	-107.4	-175.5 (PbI_2)

Supplementary Table 2. The conductivity and work function of Ag and Bi. The conductivity of 100 nm Ag and Bi metal film was measured by Four-probe Resistivity Tester.

**Metal**	**conductivity（S m^{-1}）**	**Work function (eV)**
**Ag**	4.45E+6	4.30
**Bi**	5.32E+4	4.25

Supplementary Table 3. Summary of the photovoltaic parameters of typical 1 cm^2 $MAPbI_3$-HPVKSCs with different thicknesses of Bi interlayers. Scanning mode: forward scan (from -0.1 V to 1.2 V).

**Thickness of Bi (nm)**	J_{SC} **(mA cm^{-2})**	V_{OC} **(V)**	**FF**	**PCE (%)**	R_S **(ohm cm^2)**	R_{SH} **(ohm cm^2)**
**0**	22.03	1.104	0.760	18.49	1.10	12394
**10**	21.92	1.101	0.758	18.29	1.13	11694
**20**	21.76	1.098	0.754	18.01	1.17	11046
**40**	21.46	1.065	0.658	15.05	2.54	6834

R_S: series resistance, R_{SH}: shunt resistance.

Supplementary Table 4. Summary of the photovoltaic parameters for the typical fresh, aged and re-prepared HPVKSCs (1 cm^2) with and without Bi interlayer, and their corresponding stabilized PCEs shown in **Supplementary Figure 10.**

**Devices**	**FS/RS**	**V_{OC} (V)**	**J_{SC} (mA cm^{-2})**	**FF**	**PCE (%)**	**$PCE_{average}$ (%)**	**PCE $_{stabilized}$ (%)**
**w/o Bi, Fresh**	FS	1.104	22.06	0.759	18.49	18.51	18.46
	RS	1.107	22.04	0.759	18.52		
**w/o Bi, Aged**	FS	1.085	20.09	0.670	14.61	14.24	14.24
	RS	1.081	18.97	0.676	13.86		
**w/o Bi, Re**	FS	1.086	20.43	0.730	16.18	15.93	15.94
	RS	1.085	19.88	0.726	15.67		
**Bi, Fresh**	FS	1.094	21.78	0.757	18.03	18.00	18.02
	RS	1.102	21.63	0.754	17.97		
**Bi, Aged**	FS	1.092	20.67	0.755	17.04	16.91	17.08
	RS	1.093	20.75	0.739	16.77		
**Bi, Re**	FS	1.094	21.52	0.741	17.44	17.50	17.45
	RS	1.093	21.45	0.741	17.55		

FS (Forward Scan): from -0.1 V to 1.2 V.
RS (Reverse Scan): from 1.2 V to -0.1 V.

Supplementary Table 5. Summary of the photovoltaic parameters for typical 1 cm^2 HPVKSCs based on different perovskites with and without Bi interlayers before and after aging, corresponding to the *J-V* curves shown in **Supplementary Figure 13** and **Supplementary Figure 17**. Scanning mode: forward scan (from -0.1 V to 1.2 V).

Aging condition	Devices	V_{OC} (V)	J_{SC} (mA cm^{-2})	FF	PCE (%)	$PCE/PCE_{initial}$
**Storing @ ambient air (without control, local climate is normally 40-90%RH)**	MA, w/o Bi, Fresh	1.102	21.93	0.763	18.43	100%
	MA, w/o Bi, Aged,1000 h	0.885	11.07	0.638	6.26	33.9%
	MA, Bi, Fresh	1.101	21.71	0.755	18.05	100%
	MA, Bi, Aged,6000 h	1.104	21.24	0.680	15.94	88.3%
**Thermal aging (85°C) @ N_2, dark, 500 h**	MA, w/o Bi, Fresh	1.101	21.88	0.759	18.28	100%
	MA, w/o Bi, Aged	1.003	11.51	0.671	7.74	42.3%
	MA, Bi, Fresh	1.098	21.80	0.748	17.92	100%
	MA, Bi, Aged	1.081	20.14	0.714	15.54	86.7%
	FAMACs, w/o Bi, Fresh	1.103	22.62	0.767	19.13	100%
	FAMACs, w/o Bi, Aged	1.051	16.81	0.662	11.69	61.1%
	FAMACs, Bi, Fresh	1.102	22.54	0.751	18.67	100%
	FAMACs, Bi, Aged	1.101	21.72	0.743	17.78	95.2%
**Light soaking @ N_2, 45°C, near MPP, 500 h**	MA, w/o Bi, Fresh	1.104	21.95	0.756	18.32	100%
	MA, w/o Bi, Aged	0.873	9.75	0.608	5.17	28.2%
	MA, Bi, Fresh	1.096	21.63	0.749	17.75	100%
	MA, Bi, Aged	1.091	20.03	0.740	16.18	91.2%
	FAMACs, w/o Bi, Fresh	1.103	22.61	0.768	19.15	100%

	FAMACs, w/o Bi, Aged	1.008	15.43	0.634	9.86	51.4%
	FAMACs, Bi, Fresh	1.109	22.45	0.752	18.72	100%
	FAMACs, Bi, Aged	1.104	21.89	0.751	18.15	96.9%

Supplementary Table 6. Comparison of the storage stability for selected HPVKSCs.

Device structure	Area (cm^2); Initial PCE (%); Encapsulation	T (℃); Humidity; Atmosphere	Stability (PCE/PCE$_{initial}$)	Ref.
TiO_2/(CsFAMA)Pb(IBr)$_3$/HSPhCN/spiro-OMeTAD/Au	1 cm^2; 19.6 %; No	20℃; n.a; Ambient air	80±1% after 6336 h	Cheng, Y.-B. et al. 2018.[4]
NiMgLiO/MAPbI$_3$/G-PCBM/CQDs/Ag	1 cm^2; 17.4 %; Yes	22℃; n.a; Ambient air	86±1% after 5000 h	Han, L. et al. 2017.[5]
PEDOT:PSS/(BA)$_2$(MA)$_3$Pb$_4$I$_{13}$/PCBM/Al	1 cm^2; 11.6 %; Yes	25℃; 65 %RH; humidity chamber	80±1% after 2250 h	Mohite, A. D. et al. 2016.[6]
PEDOT:PSS/MAPbI$_3$/PCBM/AZO/SnO$_x$/Ag	0.018 cm^2; 11 %; No	23℃; 50 %RH; Ambient air	100±1% after 350 h	Riedl, T. et al. 2017.[7]
SnO_2/(CsFAMA)Pb(IBr)$_3$/spiro-OMeTAD/PBDB-T/Au	0.09 cm^2; n.a.; No	22.5±2.5℃; 25 %RH; Ambient air	90±1% after 3900 h	Li, G. et al. 2018.[8]
NiMgLiO/MAPbI$_3$/PCBM/BCP/Bi/Ag	1 cm^2; 18.05 %; No	25±5℃ 65±25%RH; Ambient air	88±1% after 6000 h	This work

Supplementary Table 7. Comparison of the thermal stability for selected HPVKSCs.

Device structure	Area (cm^2); Initial PCE (%); Encapsulation	T (°C); Humidity; Atmosphere	Stability ($PCE/PCE_{initial}$)	Ref.
NiMgLiO/$MAPbI_3$/ G-PCBM/CQDs/Ag	1 cm^2; 15.3 %; Yes	85°C; 50 %RH; Ambient air	98 ±1% after 500 h	Han, L et al. 2017.[5]
TiO_2/(CsFAMA)Pb(IBr)/ HSPhCN/spiro-OMeTAD/Au	1 cm^2; 17.5 %; Yes	60°C; n.a.; Ambient air	70 ±1% after 384 h	Cheng, Y.-B. et al. 2018.[4]
PEDOT:PSS/$MAPbI_3$/PCBM/AZO/SnO_x/Ag	0.018 cm^2; 12 %; No	60°C 0 %RH; N_2	100 ±1% after 1000 h	Riedl, T. et al. 2017.[7]
NiO/(FACs)$Pb(IBr)_3$ /LiF/PCBM/SnO_2/ZTO/ITO/LiF/Ag	0.12 cm^2; 10 %; Yes	85°C; 85 %RH; humidity chamber	100 ±1% after 1000 h	McGehee, M.D. et al. 2017.[9]
NiMgLiO/$MAPbI_3$/ PCBM/BCP/Bi/Ag	1 cm^2; 18.0 ±0.2 %; No	85°C 0 %RH; N_2	86 ±1% after 500 h	This work
NiMgLiO/ (FAMACs)$Pb(IBr)_3$/ PCBM/BCP/Bi/Ag	1 cm^2; 18.7 ±0.3 %; No	85°C 0 %RH; N_2	95 ±1% after 500 h	This work

Supplementary Table 8. Comparison of the operational stability for selected HPVKSCs.

Device structure	Area (cm^2); Initial PCE (%);	Aging Temperature and Illumination	Tracking Time (h)	PCE Change in Dark (%)	Estimated T_{S80}[a] (h)	Ref.
c-TiO_2/TiO_2/(RbFAMACs)$Pb(IBr)_3$/spiro-OMeTAD/Au	1 cm^2; 17 %;	85℃, white LED, 100 mW cm^{-2}	500 h	+5%	2,000 h	Saliba, M. et al. 2016.[10]
Cl-TiO_2/(FAMACs)$Pb(IBr)_3$/spiro-OMeTAD/Au	1.1 cm^2; 20 %;	room temperature, full solar spectrum, 100 mW cm^{-2}	500 h	+10%	3,000 h	Tan, H. et al. 2017.[11]
PTAA/(FAMACs)$Pb(IBr)_3$/PS/C60/Cu	1 cm^2; 16 %;	room temperature, white LED, 100 mW cm^{-2}	170 h	n.a.	9,000 h	Stolterfoht, M. et al. 2017.[12]
NiO/(FACs)$Pb(IBr)_3$/LiF/PCBM/SnO_2/ZTO/ITO/LiF/Ag	0.12 cm^2; 13 %	35℃, full solar spectrum, 100 mW cm^{-2}	1000 h	n.a.	∞	McGehee, M.D. et al. 2017.[9]
NiO/(CsMA)PbI_3/PCBM/BCP/AZO/Ag/Al_2O_3	0.1 cm^2; 16.5 %	85℃, 1 Sun illumination, 100 mW cm^{-2}	1000 h	n.a.	1,500h	Park, N.-G. *et al.* 2018.[13]
NiMgLiO/$MAPbI_3$/PCBM/BCP/Bi/Ag	1 cm^2; 17.75%	45℃, white LED, I_J[b]	500 h	n.a.	2,000 h	This work
NiMgLiO/(FAMACs)$Pb(IBr)_3$/PCBM/BCP/Bi/Ag	1 cm^2; 18.72%	45℃, white LED, I_J[b]	500 h	n.a.	11,800 h	This work

a, T_{S80}: The time at which the device has degraded to 80% of the initial efficiency. T_S80 can be extracted from the MPPT.[14]

b, I_J: The light intensity was calibrated to achieve the same J_{SC} of PVKSCs as upon AM1.5G solar irradiation.

Supplementary Note 1.

According to a recent density function theory (DFT)-based computation study, the formation energy of Au^+ in $MAPbI_3$ is low,[15] but the Au^+ can't be observed in experiment.[16] Actually, a stable product of AuI is hard to gain by the directly reaction between iodine and golden. As reported, AuI can be formed by heating Au power and iodine (1:1.5 mol) in a closed ampoule for > 4 days at exactly 393±3 ℃.[17] Only in special iodine-iodide solution, the reaction between iodine and golden can be easy to occur, and their resultant complex compounds are more stable than the product of solid-solid reaction.[18]

Supplementary References

1. W.M. Haynes, *CRC handbook of chemistry and physics*, CRC press, 2014.
2. Cubicciotti D. Enthalpy of Formation of bismuth (III) Iodide and the Dissociation Energy of bismuth (I) Iodide. *Inorganic Chemistry* **7**: 211-213 (1968).
3. Chase, M. W. *et al.* JANAF thermochemical tables. *J. Phys. Chem. Ref. Data* **3**, 311-480 (1974).
4. Lu, J. *et al.* Interfacial benzenethiol modification facilitates charge transfer and improves stability of cm-sized metal halide perovskite solar cells with up to 20 % efficiency. *Energy Environ. Sci.* **11**, 1880-1889 (2018).
5. Bi, E. *et al.* Diffusion engineering of ions and charge carriers for stable efficient perovskite solar cells. *Nat. Commun.* **8**, 15330 (2017).
6. Tsai, H. *et al.* High-efficiency two-dimensional Ruddlesden–Popper perovskite solar cells. *Nature* **536**, 312-316 (2016).
7. Brinkmann, K. O. *et al.* Suppressed decomposition of organometal halide perovskites by impermeable electron-extraction layers in inverted solar cells. *Nat. Commun.* **8**, 13938 (2017).
8. Qin, P.-L. *et al.* Stable and efficient organo-metal halide hybrid perovskite solar cells via π-conjugated lewis base polymer induced trap passivation and charge extraction. *Adv. Mater.* **30**, 1706126 (2018).
9. Bush, K. A. *et al.* 23.6%-efficient monolithic perovskite/silicon tandem solar cells with improved stability. *Nat. Energy* **2**, 17009 (2017).
10. Saliba, M. *et al.* Incorporation of rubidium cations into perovskite solar cells improves photovoltaic performance. *Science* **354**, 206-209 (2016).
11. Tan, H. *et* al. Efficient and stable solution-processed planar perovskite solar cells via contact passivation. *Science* **355**, 722-726 (2017).
12. Stolterfoht, M. *et al.* Approaching the fill factor Shockley–Queisser limit in stable, dopant-free triple cation perovskite solar cells. *Energy Environ. Sci.* **10** 1530-1539 (2017).
13. Seo, S., Jeong, S., Bae, C., Park, N.-G. & Shin, H. Perovskite solar cells with

inorganic electron-and hole-transport layers exhibiting long-term (≈ 500 h) stability at 85 ℃ under continuous 1 sun illumination in ambient air. *Adv. Mater*. **30**, 1801010 (2018).

14. Saliba, M. *et al*. Measuring aging stability of perovskite solar cells. *Joule* **6**, 1019-1024 (2018).
15. Ming, W., Yang, D., Li, T., Zhang, L. & Du, M.-H. Formation and diffusion of metal impurities in perovskite solar cell material $CH_3NH_3PbI_3$: implications on solar cell degradation and choice of electrode. *Adv. Sci.* **5**, 1700662 (2018).
16. Zhao, L. et al. Redox chemistry dominates the degradation and decomposition of metal halide perovskite optoelectronic devices. *ACS Energy Lett.* **1**, 595–602 (2016).
17. Dietrich, B., Herrmann, W. A. & Hiller, W. Synthetic Methods of Organometallic and Inorganic Chemistry, Thieme press, 60-61 (1996).
18. Tran, T. & Davis, A. Gold dissolution in iodide electrolytes. *Hydrometallurgy* **125**, 69-75, (2012).
