# Supplementary material for: A chemically inert bismuth interlayer enhances long-term stability of inverted perovskite solar cells
Source: Nat Commun. 2019 Mar 11;10:1161. doi: 10.1038/s41467-019-09167-0 (PMC6411982; doi:10.1038/s41467-019-09167-0)
Supplement: Supplementary file 3 — Solar Cells Reporting Summary [file 41467_2019_9167_MOESM3_ESM.pdf]

nature research

Corresponding author(s): Wei Chen

Solar Cells Reporting Summary

Nature Research wishes to improve the reproducibility of the work that we publish. This form is intended for publication with all accepted papers reporting the characterization of photovoltaic devices and provides structure for consistency and transparency in reporting. Some list items might not apply to an individual manuscript, but all fields must be completed for clarity.

For further information on Nature Research policies, including our data availability policy, see Authors & Referees.

Experimental design

Please check: are the following details reported in the manuscript?

1. Dimensions

Item	Yes	No	Details
Area of the tested solar cells	☒	☐	Methods
Method used to determine the device area	☒	☐	Methods

2. Current-voltage characterization

Item	Yes	No	Details
Current density-voltage (J-V) plots in both forward and backward direction	☒	☐	Figure 3 ,Figure 5
Voltage scan conditions<br>*For instance: scan direction, speed, dwell times*	☒	☐	Figure 3 ,Figure 5
Test environment<br>*For instance: characterization temperature, in air or in glove box*	☒	☐	Methods
Protocol for preconditioning of the device before its characterization	☒	☐	Methods
Stability of the J-V characteristic<br>*Verified with time evolution of the maximum power point or with the photocurrent at maximum power point; see ref. 7 for details.*	☒	☐	Figure 3

3. Hysteresis or any other unusual behaviour

Item	Yes	No	Details
Description of the unusual behaviour observed during the characterization	☒	☐	Figure 5
Related experimental data	☒	☐	Supplementary Figure 12,Supplementary Figure 15,Supplementary Figure 16,Supplementary Figure 18,Supplementary Figure 19

4. Efficiency

Item	Yes	No	Details
External quantum efficiency (EQE) or incident photons to current efficiency (IPCE)	☒	☐	Supplementary Figure 7
A comparison between the integrated response under the standard reference spectrum and the response measure under the simulator	☒	☐	Supplementary Figure 7
For tandem solar cells, the bias illumination and bias voltage used for each subcell	☐	☒	*Explain why this information is not reported/not relevant.*

5. Calibration

Item	Yes	No	Details
Light source and reference cell or sensor used for the characterization	☒	☐	Methods
Confirmation that the reference cell was calibrated and certified	☒	☐	Methods

Calculation of spectral mismatch between the reference cell and the devices under test	☒ Yes ☐ No	*State where this information can be found in the text.*

6. Mask/aperture

Size of the mask/aperture used during testing	☒ Yes ☐ No	Methods
Variation of the measured short-circuit current density with the mask/aperture area	☒ Yes ☐ No	Methods

7. Performance certification

Identity of the independent certification laboratory that confirmed the photovoltaic performance	☐ Yes ☒ No	No certification
A copy of any certificate(s) *Provide in Supplementary Information*	☐ Yes ☒ No	No certification

8. Statistics

Number of solar cells tested	☒ Yes ☐ No	Figure 6
Statistical analysis of the device performance	☒ Yes ☐ No	Figure 6

9. Long-term stability analysis

Type of analysis, bias conditions and environmental conditions *For instance: illumination type, temperature, atmosphere humidity, encapsulation method, preconditioning temperature*	☒ Yes ☐ No	Stability test of the devices
